# Living myocardial slices for the study of nucleic acid-based therapies

**DOI:** 10.3389/fbioe.2023.1275945

**Published:** 2023-10-24

**Authors:** R. Nunez-Toldra, A. Del Canizo, I. Secco, L. Nicastro, M. Giacca, C. M. Terracciano

**Affiliations:** ^1^ National Heart and Lung Institute, Imperial College London, London, United Kingdom; ^2^ King’s College London, School of Cardiovascular Medicine and Sciences, London, United Kingdom

**Keywords:** living myocardial slices, cardiac gene therapy, adeno-associated viruses, cardiac electrophysiology, cardiac tissue

## Abstract

Gene therapy based on viral vectors offers great potential for the study and the treatment of cardiac diseases. Here we explore the use of Living Myocardial Slices (LMS) as a platform for nucleic acid-based therapies. Rat LMS and Adeno-Associated viruses (AAV) were used to optimise and analyse gene transfer efficiency, viability, tissue functionality, and cell tropism in cardiac tissue. Human cardiac tissue from failing (dilated cardiomyopathy) hearts was also used to validate the model in a more translational setting. LMS were cultured at physiological sarcomere length for 72-h under electrical stimulation. Two recombinant AAV serotypes (AAV6 and AAV9) at different multiplicity of infection (MOI) expressing enhanced green fluorescent protein (eGFP) were added to the surface of rat LMS. AAV6 at 20,000 MOI proved to be the most suitable serotype without affecting LMS contractility or kinetics and showing high transduction and penetrability efficiency in rat LMS. This serotype exhibited 40% of transduction efficiency in cardiomyocytes and stromal cells while 20% of the endothelial cells were transduced. With great translational relevance, this protocol introduces the use of LMS as a model for nucleic acid-based therapies, allowing the acceleration of preclinical studies for cardiac diseases.

## 1 Introduction

With now affecting 30% of the global population, cardiovascular diseases are one of the first causes of morbidity and mortality worldwide. With an increase in life expectancy and in the prevalence of associated risk factors, this incidence is only expected to rise (Savarese and Lund, 2017; Groenewegen et al., 2020). Despite efforts to improve treatment, prognosis remains poor, such as 5-year survival rate currently at 50% after heart failure (Taylor et al., 2017). The only standard therapy addressing the irreversible failure or loss of functional cardiomyocytes is cardiac transplantation, limited by donor availability and the need for life-long immunosuppression. Gene therapy using viral vectors offers great potential for the study and the treatment of cardiac diseases such as heart failure or monogenic heart diseases, in particular genetic cardiomyopathies. Existing treatment is limited to symptomatic patients with recognised disease and adverse ventricular remodelling. Cardiac gene therapy would target early effects of genetic and environmental triggers, ideally before disease has even developed (Tzahor and Poss, 2017; Liu et al., 2020).

Considering all the available viral gene delivery systems, adeno-associated virus (AAV) vectors are attractive candidates for cardiovascular gene transfer because of their efficient transfer and stable expression of target genes in specific post-mitotic tissues, such as brain, liver or heart (Zincarelli et al., 2008; Prasad et al., 2011). In non-proliferative differentiated cells, such as cardiomyocytes, recombinant AAVs can provide persistent expression which can last at least up to 10 years (Bulcha et al., 2021). Although, these viruses do have the ability to infect humans, they are devoid of significant pathogenicity and do not elicit a major immune response (Wang et al., 2019; Li and Samulski, 2020; Bulcha et al., 2021). Clinical trials with AAV-based therapies currently account for approximately 30% of all virus-based clinical trials, with a total of 240 registered up to date (Bulcha et al., 2021). Transduction efficiency of AAVs in different tissues depends mainly on their capsid serotype. Each serotype displays different surface antigens that are recognized by host cell receptors. The optimization of cardiac tropism and the transduction efficacy will be essential for designing an AAV-based gene therapy targeting heart failure. Nevertheless, there is inconsistent literature on the efficiency of the 13 known AAVs serotypes for cardiac tissue transduction due to different routes of administration, viral doses and animal models, affecting the transduction efficiency (Zincarelli et al., 2010; Katz et al., 2017). Although, different studies indicate that AAV1, 6, 8, and 9 were the most cardiotropic serotypes (Inagaki et al., 2006; Palomeque et al., 2007; Bish et al., 2008; Zincarelli et al., 2010; Gabisonia et al., 2019; Liu et al., 2020), more evidence is required to determine the ideal AAV conditions for gene therapy in cardiac disease.

The value of living myocardial slices (LMS) as an emergent experimental model that establishes an intermediate point between isolated cells and animal models is now widely recognized (Fischer et al., 2019; Ou et al., 2019; Liu et al., 2020; Pitoulis et al., 2020; Meki et al., 2021). LMS are ultra-thin slices of ventricular myocardium that can be obtained from small and large animal hearts (Watson et al., 2017). Their multicellular nature makes them more representative than standard cardiac research platforms such as isolated cardiomyocytes or human induced pluripotent-derived cardiomyocytes.

After years of experience working with this platform, our group published a protocol for generating and culturing LMS. This protocol is the result of a meticulous optimisation to achieve high levels of cell viability ensuring that around 60%–80% of cardiomyocytes remain intact (Watson et al., 2017; Nunez-Toldra et al., 2022). We have demonstrated that culturing LMS under biomimetic electromechanical stimulation by applying electrical pacing and mechanical load to ensure physiological sarcomeric length is essential for maintaining viability in culture (Watson et al., 2017; Pitoulis et al., 2021). This statement has been further corroborated by two other recent studies from other laboratories (Fischer et al., 2019; Qiao et al., 2019). Rat, rabbit, dog and human LMS have been previously generated by our group and proven to remain viable in culture. Measuring contractility at real time, LMS cultured at physiological load, have been proven to remain viable in culture for up to 72 h without changes in contractility (Pitoulis et al., 2020; Nunez-Toldra et al., 2022). In addition, a protocol has been validated to obtain a model of cardiac fibrosis-remodelling in LMS by applying pathological mechanical load (Nunez-Toldra et al., 2022).

Up to date, no more than three studies have reported the use of heart slices as a platform for nucleic acid-based therapies (Kang et al., 2016; Ou et al., 2019; Liu et al., 2020). Surprisingly, AAVs have only been used as delivery vectors in one of these studies (Liu et al., 2020). In such study, the technique used to obtain the slices was poorly optimised and there was no biomimetic electromechanical stimulation applied, and LMS contractility was not assessed. In fact, the authors themselves acknowledged that the slices in this study lost 25% of the cardiomyocytes after the first day of culture and presented ischaemic-like tissue morphologies (Liu et al., 2020). Both observations lead to the assumption that functionality of these slices was probably altered. Therefore, reliable studies on AAV transduction of functional LMS are needed to explore its application as a potential platform for studying nucleic acid-based therapies.

The general aim of this study was to evaluate and optimise the use of LMS as a platform for nucleic acid-based therapies. Rat LMS and different AAV were used to analyse gene transfer efficiency, viability, tissue functionality, and cell tropism in cardiac tissue. Human cardiac samples from failing (dilated cardiomyopathy) hearts were also used to validate the model in a more translational setting.

## 2 Materials and methods

### 2.1 AAVs

**FIGURE 1:**
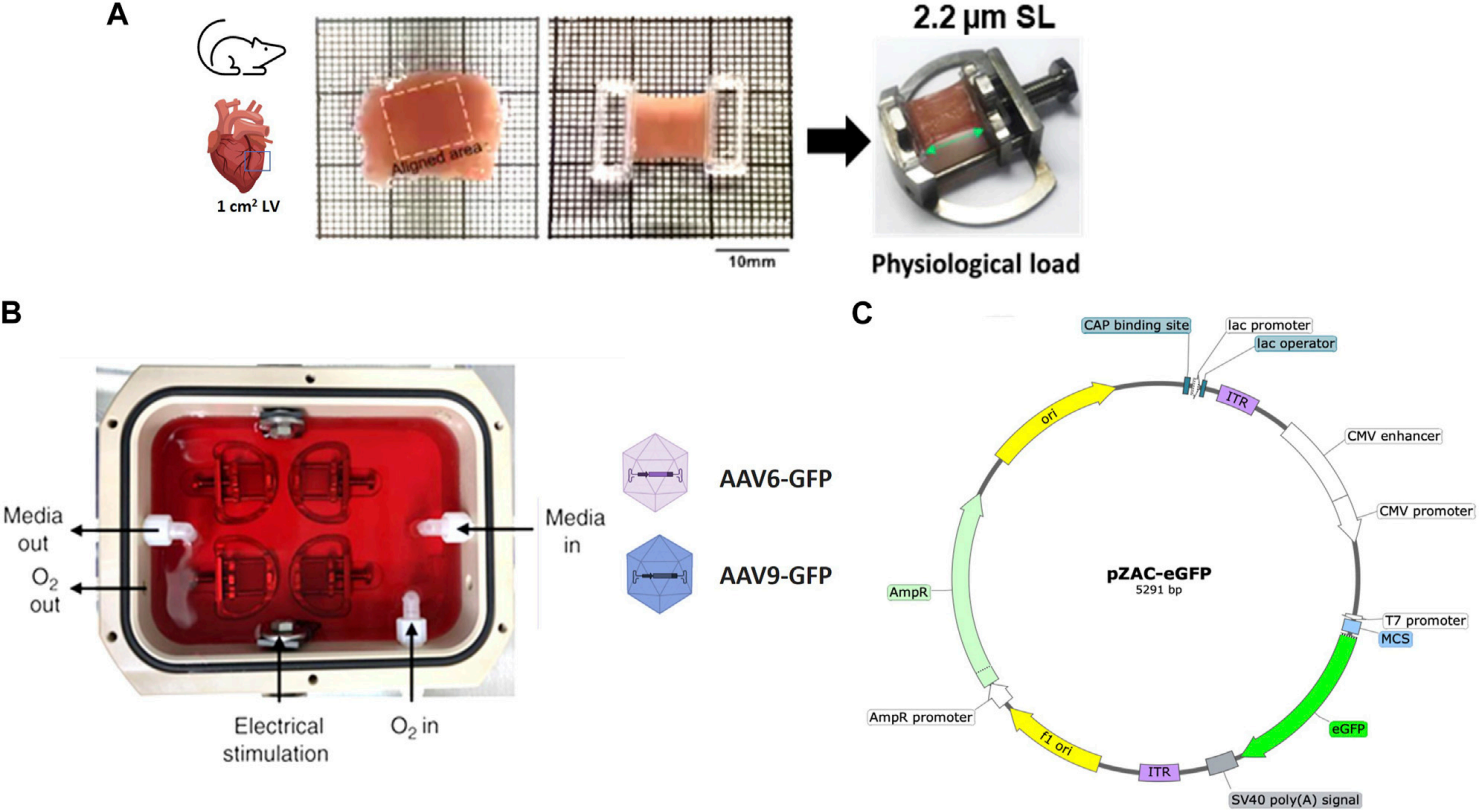
Preparation and AAV transduction of living myocardial slices (LMS). **(A)** 300 µm LMS from LV myocardium were prepared, glued to PTFE-coated holders, and mounted on custom stretchers. **(B)** LMS were cultured at physiological sarcomere length (2.2 µm SL) for 72-h under electrical stimulation. Two recombinant AAV serotypes (AAV-6 and AAV-9) at different MOI expressing eGFP were added to the surface of the LMS. **(C)** pZAC-eGFP plasmid map.

Recombinant AAV vectors were prepared by the AAV Vector Unit at the ICGEB Trieste (www.icgeb.org/avu-core-facility) according to established procedures (Arsic et al., 2003). Briefly, AAV vectors of serotypes 6 and 9 were generated in HEK293T cells, using a triple-plasmid co-transfection for packaging. Viral stocks were obtained by PEG precipitation and two consequent CsCl gradient centrifugations, which resulted in less than 5% contamination of empty particles. Titration of AAV viral particles was performed by real-time PCR quantification of the number of viral genomes, measured as constitutive cytomegalovirus (CMV) DNA copy number. The viral preparations had titers between 1 × 10^13^ and 1 × 10^14^ viral genomes (vg) per ml. The AAV plasmid vector map is shown in Figure 1C. Expression of the transgene is under the control of the CMV immediate early promoter.

### 2.2 Preparation of LMS

All animal procedures were performed under license by the UK Home Office, in agreement with the United Kingdom Animals (Scientific Procedures) Act 1986. Animals were sacrificed under isoflurane-induced anaesthesia (4% isoflurane at 4 L/min oxygen) by cervical dislocation in accordance with the guidelines established by the European Directive on the protection of animals used for scientific purposes (2010/63/EU).

LMS were obtained from Sprague-Dawley male rats (300–350 g). After the sacrifice of the rat, the heart and the surrounding tissues were excised, immersed in hot Tyrode’s solution (37°C) slicing solution containing 1000 IU/mL of heparin and immediately transferred into cold (4°C) slicing solution. The slicing solution was composed of cold Tyrode’s solution, containing 2,3-Butanedione Monoxime 30 mM, NaCl 140 mM, KCl 9 mM, Glucose 10 mM, HEPES 10 mM, MgCl2 1 mM, and CaCl2 1 mM dissolved in distilled water and adjusted to pH 7.4.

The left ventricular tissue block was then prepared by first removing surrounding tissues along with the atria and the right ventricle, and then incising the septum in the direction of the apex to open the left ventricle. The tissue was then mounted on top of a 4% agarose block using a topical skin adhesive (Histoacryl ^®^ Octyl Micro, Braun Surgical S.A), placing the epicardium on the underside. The agarose block was glued to a metallic specimen holder that was placed into an organ bath filled with cold Tyrode’s solution and bubbled with filtered 100% oxygen. The bath was attached to a Vibrating Microtome 7,000 (Campden Instruments Ltd., United Kingdom). The blade alignment was adjusted to a *z*-axis error of <1.0 µm before each use. Slicing was performed at 2 mm amplitude, 0.03 mm/s feed rate, 80 Hz, and section thickness of 300 µm. About four to six slices were obtained per heart.

Once a slice was detached from the block, it was transferred to a Petri dish. The orientation of the fibres was visualised with an optical microscope to identify and cut an area of approximately 1 cm^2^ of fibres alignment. This area was isolated by trimming with a razor blade, and two custom-made 3D printed T-Glase rectangular holders were attached perpendicular to the fibres along the cross-sectional area of the slice were glued to each side. These rings allowed the slice to be placed on custom-made stainless-steel stretchers that enabled the slice to be stretched as much as desired. The protocol is outlined in Figure 1A and Supplementary Figure S1.

Human samples were provided by the NIHR Cardiovascular Biomedical Research Unit at the Royal Brompton and Harefield NHS Foundation Trust and Imperial College London. The study performed was approved by a UK institutional ethics committee (NRES ethics number for biobank samples: 09/H0504/104 + 5; Biobank approval number: NP001-06-2015 and MED_CT_17_079) and Imperial College London. Informed consent was obtained from each patient/family involved in this study. Human failing hearts (dilated cardiomyopathy) were perfused with cold cardioplegia solution and arrested *in situ* prior to being explanted. The specimens were placed in cold cardioplegia, placed on ice, and transported to the laboratory. A 1.5 cm^2^ tissue block was dissected out of the left ventricular by making an incision through the full thickness of the free wall. The tissue was mounted epicardial surface down and sliced in the same manner as rat LMS.

### 2.3 Culture and transduction of LMS

LMS were cultured for 72 h. To minimise tissue damage, it is essential to culture the LMS mimicking *in vivo* physiological conditions by the application of a constant mechanical and electrical stimulation (Watson et al., 2017). For mechanical stimulation, slices were stretched to an average sarcomeric length of 2.2 µm (17.5% stretch) described as physiological sarcomere stretch. All length measurements were taken with callipers. Stretched LMS were then placed in groups of four in custom-made culture chambers filled with 120 mL of culture media (Figure 1B; Supplementary Figure S1).

Culture media consisted of medium-199 with Earl’s salts (Sigma Aldrich, United Kingdom) supplemented with 3% Penicillin-Streptomycin (Sigma Aldrich), 0.1% Insulin Transferrin Selenium (Sigma Aldrich), 10% FBS, adrenaline 4 nM (Sigma Aldrich), noradrenaline 4 nM (Sigma Aldrich), triiodothyronine 2.15 nM (Sigma Aldrich), dexamethasone 100 nM (Sigma Aldrich), endothelial cell growth supplement 7.5 μg/mL, and ascorbic acid 0.02 mg/mL (Sigma Aldrich). For electrical stimulation, a constant field stimulation of 15 V voltage, 1.0 Hz frequency and 10 m width was applied to the culture chamber.

For viral transduction of the slices, a drop of 15 µL of viral particles was placed on top of each slice. The transduction concentrations used were either 10,000 or 20,000 MOI. To calculate the volume to be added, each slice was considered to have approximately 1 million cells (43). The chamber was then closed and placed in an incubator at 37°C. During culture, media was perfused with 95% O2 + 5% CO2 at a rate of 0.25 L/min. One hour after addition of the virus, continuous media circulation at a rate of 15 mL/min was activated to maintain a constant O^2^/CO^2^ concentration throughout the chamber.

After 24 h, 50 mL of fresh media was added to the chamber to prevent evaporation. 48 h after the start of culture 50 mL of the used media was removed to eliminate metabolites that can negatively affect the culture, and further 50 mL of fresh media were added. The culture was terminated after 72 h.

### 2.4 Contractility of LMS

LMS contractility data were acquired with an HSE-HA isometric force transducer F30 (Harvard Apparatus, United States). 72 h after culture, each LMS was removed from their chamber and stretcher, and placed in a Petri dish filled with culture media preheated to 37 C. One of the holders on the side of the slice was attached to a hook and the other one to the spring hook of the force transducer. The slices were gradually stretched until maximum amplitude of contractility was obtained and subjected to a constant field stimulation of 30–40 V voltage, 1.0 Hz frequency and 30–40 m width. Data were recorded and analysed using AxoScope software (Molecular Devices LLC, United States).

### 2.5 Cell viability assay

The viability of cultured LMS was assessed using CellTiter 96 Aqueous One solution cell proliferation assay (Promega, Southampton, United Kingdom), following manufacturer’s instructions. Briefly, a 2 mm diameter sample was obtained from the cultured LMS using a biopsy puncher. The sample was incubated for 20 min at 37°C, 95% O2 5% CO2 in a 96-well plate filled with 100 μL M-199 + 40 μL CellTiter96. The absorbance of the media in the well was then recorded immediately at 490 nM using a 96-well plate reader (Labtech, TX, United States). Each value was normalized by subtracting the background (LMS media + CellTiter96) absorbance.

### 2.6 Immunofluorescence staining of LMS

Slices were washed in DPBS and fixed in 4% paraformaldehyde (PFA; Brand, Country) for 15 min while placed in the stretchers. To permeabilise and block non-specific binding sites, slices were incubated with 1.5% TritonTM X-100 (Sigma Aldrich), 5% bovine serum albumin (BSA), 10% foetal bovine serum (FBS) and 5% horse serum in DPBS for 3 h at RT. Slices were then incubated overnight at 4°C with the primary antibodies (Supplementary Table S1) in 1% BSA. After three 30-min washes with DPBS, they were incubated with the respective secondary antibodies for 2 h at RT and washed three more times in 30-min washes with DPBS. Then, to stain the nuclei, slices were incubated with Hoechst 33,342 (1:1,000) for 15 min at RT and washed again as above.

Confocal microscopy was performed using a LSM-780 inverted confocal laser scanning microscope (Zeiss, Germany) and the ZEN Imaging Software (Zeiss). Image analysis was performed with ImageJ (National Institutes of Health, United States) quantifying pixels with the auto-threshold tool. The JACoP plugin for colocalization analysis was used to obtain the Manders’ Coefficient M2 (Bolte and Cordelieres, 2006). For each LMS at least five z-stack images from ×10 and ×20 magnification from different sections were considered.

To assess the penetrability of the transduction, 15 µm transversal sections of the slices were obtained using a Cryotome^©^ Standard (Thermo Fisher Scientific, United States). For this purpose, before sectioning, the LMS were fixed in 4% PFA and placed in 15% sucrose in distilled water until the tissue sank, and subsequently in 30% sucrose. Cryosections were immunostained following a similar protocol to that of the myocardial slices.

### 2.7 Statistical analysis

All statistical analysis was performed with Prism 9 (GraphPad, US). Either a 1- or 2-way ANOVA with a Tukey’s *post hoc* test for multiple comparisons were performed for all experiments. Significance was defined as: non-significant: *p* > 0.05; *: *p* < 0.05, **: *p* < 0.01, ***: *p* < 0.001, ****: *p* < 0.0001. All data are expressed as the group mean and error bars indicate standard error of mean. For all experiments the number of LMS is represented in each scatter dot plot graph where each dot represents an individual LMS and N the number of biological replicates/animals.

## 3 Results

### 3.1 Transduction efficiency and penetrability of AAV6/9-GFP in LMS

Rat LMS were transduced at different MOIs with either AAV6 or AAV9 carrying a pZAC-eGFP vector. 72 h after transduction, changes in LMS viability and contractility were assessed.

**FIGURE 2:**
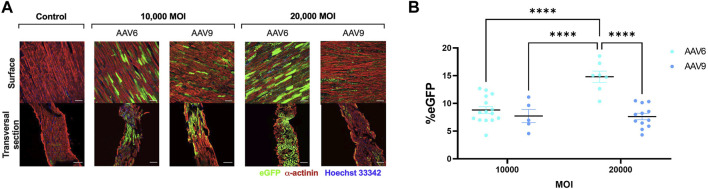
Efficiency of AAV transduction in rat LMS. **(A)** Representative images of the surface and transversal sections of LMS stained for enhanced green fluorescence protein (eGFP), α-actinin, and Hoechst 33,342 transduced with either AAV6-GFP or AAV9-GFP at 10,000 or 20,000 MOI. Scale bars: 100 µm. **(B)** Quantification of eGFP expression expressed as percentage of area positive for eGFP. 3 images were taken from different regions of each LMS. Each dot represents a LMS (10,000 MOI AAV6 N:5; 10,000 MOI AAV9 N:3; 20,000 AAV6 N:4; 20,000 MOI AAV9 N:5). N: Number of biological replicates. ****p < 0.0001.

EGFP expression was detectable after transduction with both AAV serotypes (Figures 2A, B). For AAV6-transduced LMS, eGFP signal was significantly higher when administered in a higher viral dose. In addition, transversal sections obtained with the cryotome revealed that complete penetrability of the tissue was also achieved at both viral concentrations after AAV6 transduction (Figure 2A). AAV9 showed penetrability of the LMS but lower transduction efficiency than AAV6 at 20,000 MOI, with no differences in eGFP expression when increasing the dose.

### 3.2 Contractility parameters of AAV6/9-GFP-transduced LMS

**FIGURE 3:**
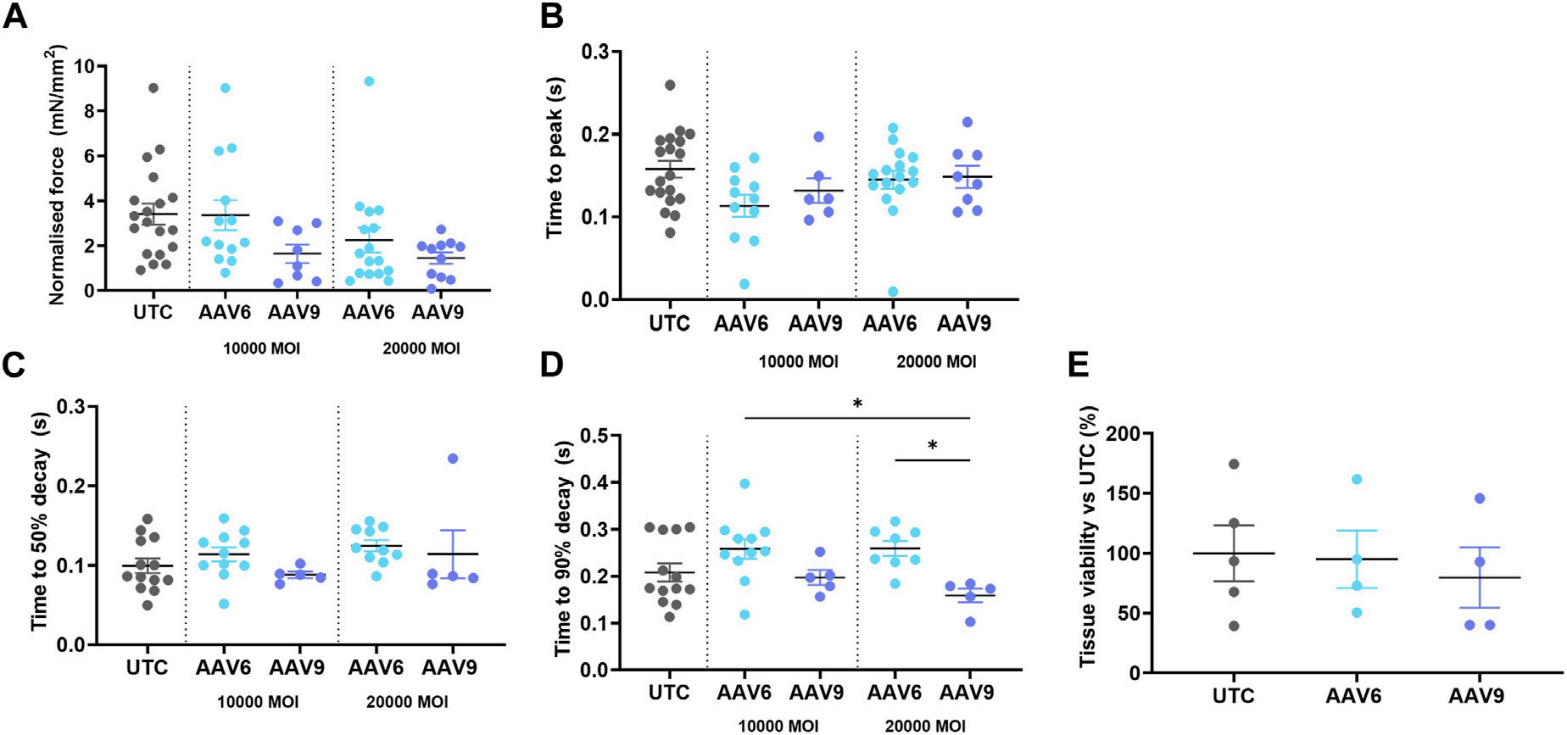
Contractility parameters and cell viability of AAV-transduced rat LMS. LMS were transduced and cultured for 72 h under electrical stimulation. The following contractility parameters were measured post-culture **(A)** Normalilsed Force *versus* cross sectional area of LMS. **(B)** Time to reach the peak amplitude of contraction. **(C)** Time between maximum amplitude of contraction and 50% of the decay. **(D)** Time between maximum amplitude of contraction and 90% of the decay. **(E)** Tissue viability post transduction *versus* untreated cells. Each dot represents a LMS (UTC N: 5-6; 10,000 MOI AAV6 N: 6; 10,000 MOI AAV9 N:4; 20,000 MOI AAV6 N:4-8; 20,000 MOI AAV9 N:4-5). N: number of biological replicates. *p < 0.05, **p < 0.01.*p < 0.05, **p < 0.01.

We then assessed whether AAV transduction produced any change in rat LMS contractility and viability and whether this was serotype- or dose-related. Results show that none of the serotypes at any of the doses used showed a significant difference in active force when compared to the non-transduced control (UTC) (Figure 3A) although values were decreased at higher MOI, especially when AAV9 was used. No differences in contractility kinetics were found except for AAV9-transduced LMS, showing a decrease in time to decay vs. AAV6 (Figures 3B–D). Finally, tissue viability was maintained after transduction with both AAV serotypes (Figure 3E) at the highest MOIs.

LMS were also obtained from the left ventricle of human explanted hearts (dilated cardiomyopathy) and transduced with AAV6-pZAC-eGFP at 20,000 MOI for 72 h under electrical stimulation. Contractility parameters were analysed 72 h post transduction showing no changes in human LMS active force or contractility kinetics after AAV6 transduction (Supplementary Figure S2).

### 3.3 Cell type-specific tropism of AAV6-GFP-transduced LMS

**FIGURE 4:**
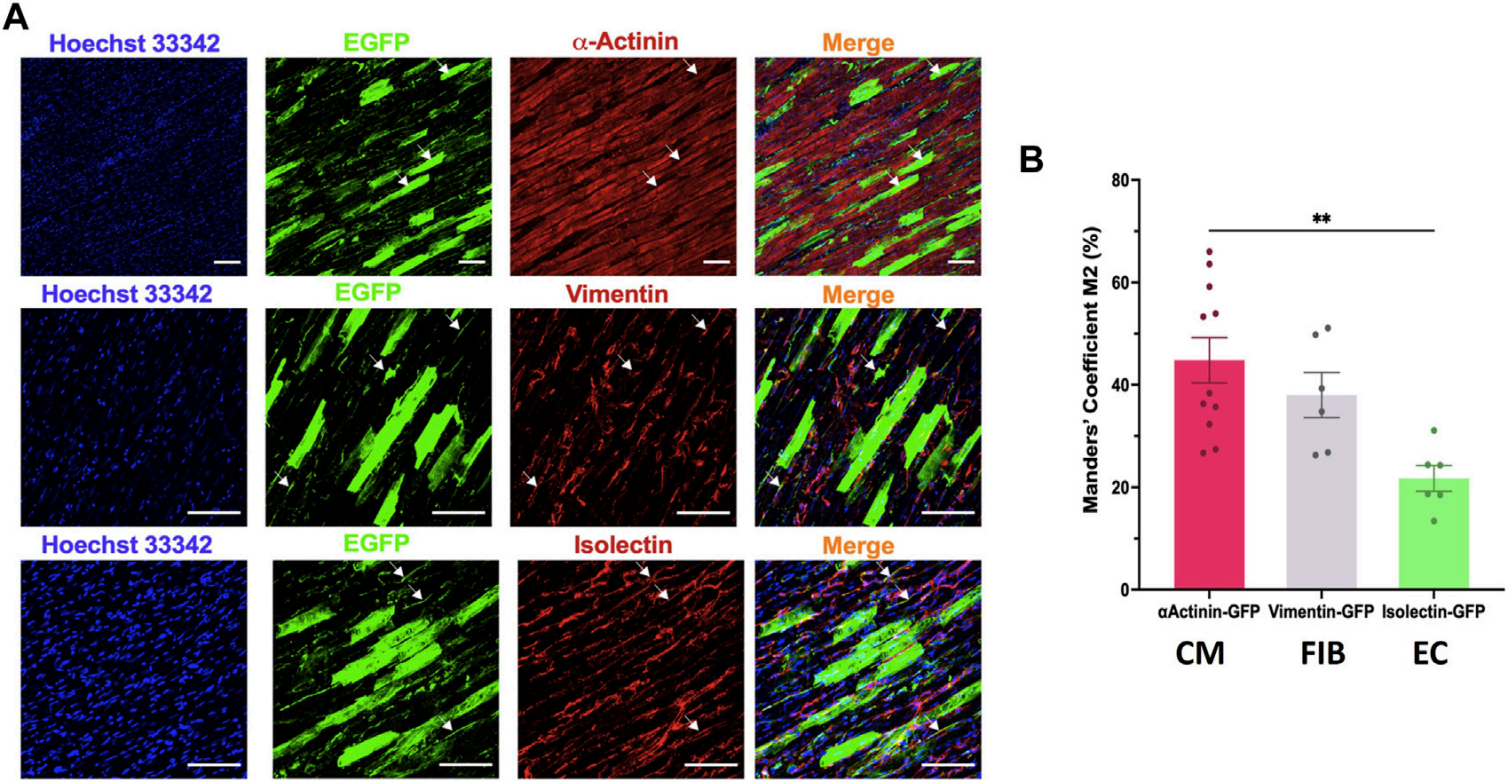
Cell tropism of AAV6 transduction in rat LMS. **(A)** Colocalization analysis of the fluorescence signal of eGFP with α-actinin (cardiomyocytes, CM), vimentin (fibroblasts, FIB), or isolectin (endothelial cells, EC) in LMS transduced with AAV6-GFP at 20,000 MOI cultured for 72 h. Scale bars: 100 µm **(B)** Quantification of the eGFP signal overlapping each of the cell specific markers [Manders’ Coefficient (Bolte and Cordelieres, 2006)]. Each dot represents a LMS (N: 3 for each staining). N: number of biological replicates. **p < 0.01.

We then analysed the cell tropism after transducing rat LMS with the most efficient condition, AAV6 at 20,000 MOI, by immunostaining with different cell markers for cardiomyocytes (α-actinin), stromal cells (vimentin) and endothelial cells (isolectin). EGFP expression was found in all cardiac cell types (Figure 4A). Manders’ Coefficient M2, which quantifies the percentage of area positive for each cell marker that is also positive for eGFP, showed a correlation of 44.80% ± 14.66% for cardiomyocytes, 38.02% ± 10.82% for stromal cells, and 21.73% ± 6.17% for endothelial cells (Figure 4B).

In addition, confocal images of human LMS transduced with AAV6-pZAC-eGFP showed successful transduction of cardiomyocytes and endothelial cells (Supplementary Figure S2).

## 4 Discussion

The treatment of cardiac diseases is undergoing a revolution, with research efforts focusing on the expansion of novel therapies such as gene therapy. However, the development of these therapies is hampered by a lack of multicellular *in vitro* platforms that reliably model cardiac physiology. A middle ground is needed between isolated cells, incapable of fully reproducing the complexity of cardiac tissue, and the use of animals, a resource that should be reduced for ethical, translational, and economic reasons. LMS have been proposed to meet this need.

In the present study, we have confirmed the feasibility of using electromechanically stimulated rat LMS to test nucleic acid-based therapies. We optimised and validated the protocol for LMS transduction using some of the most cardiotropic AAVs and evaluating both efficiency and viability at electrophysiologic level.

### 4.1 AAV6 shows the highest transduction and penetrability efficiency

AAV vectors expressing the eGFP transgene under a CMV promoter were used to validate the use of LMS for AAV-delivered therapies. To our knowledge, this is the first study in doing so.

After eGFP transductions, we found AAV6 to be more efficient than AAV9. We have demonstrated that AAV6 not only transduces superficial layers but successfully penetrates through the entire thickness of the slice. While AAV9 at a lower dose also shows adequate penetrability, a lower level of penetrability and lower cardiomyocyte transfection are noticeable in the images when administered at a higher dose. Previous studies in mice and pigs comparing the same AAV serotypes also confirm the superiority of AAV6 to transduce cardiac tissue (Zincarelli et al., 2010; Gabisonia et al., 2019). However, this comparison has limited translational relevance, as the efficacy of each serotype has proven to be highly dependent on the animal model, dose and administration method used (Katz et al., 2017; Bass-Stringer et al., 2018).

In addition, these results proved that the transduction of LMS with AAV6 using a CMV promoter does not affect cell viability or contractility parameters at any of the indicated concentrations, confirming the suitability of this serotype for future experiments. On the other hand, treatment with AAV9 at high doses (20,000 MOI) did influence contractility kinetics of LMS, making the tissue contract faster. It is important to study this effect further because it could pose a safety concern for the use of this serotype in clinics.

### 4.2 AAV6 shows cell type-specific tropism in cardiac tissue

Cell type-specific tropism of viral vectors is of great importance for their translational application. The multicellularity of LMS make them a very convenient platform for these studies. Hence, cell tropism of AAV6, the most efficient serotype from previous data, was evaluated. Results suggest that AAV6 transduced approximately 40% of cardiomyocytes and stromal cells, as well as a smaller proportion of endothelial cells (20%). Interestingly, Liu et al. report their AAV6 transduction of mice LMS to achieve 100% of cardiomyocytes and 12.5% of stromal cells (Liu et al., 2020). We believe that these inconsistencies could be due to differences in quantification methods, the setting of a different threshold and different final phenotype of the slices.

Nucleic acid-based transduction of human heart tissue could be considered a valid and translational alternative to the use of transgenic animal models. During the development of this study, we had access to limited human failing hearts. LMS from the left ventricle were produced and transduced with AAV6-pZAC-eGFP. These preliminary data showed transduction in human cardiomyocytes, cardiac fibroblasts, and endothelial cells without changes in contractility parameters. Although further studies must be done to better characterise AAV transduction in human LMS, these results encourage the use of LMS as a convenient preclinical model.

This research project brings the use of LMS as a platform for nucleic acid-based therapies in animal studies. Moreover, with great translational relevance, opens the possibility to adapt the AAV transduction protocol in human LMS allowing the acceleration of preclinical studies of gene therapy in cardiac diseases.

## Data Availability

The original contributions presented in the study are included in the article/Supplementary Material, further inquiries can be directed to the corresponding authors.

## References

[B1] Arsic N. Zentilin L. Zacchigna S. Santoro D. Stanta G. Salvi A. (2003). Induction of functional neovascularization by combined VEGF and angiopoietin-1 gene transfer using AAV vectors. Mol. Ther. 7 (4), 450–459. 10.1016/s1525-0016(03)00034-0 12727107

[B2] Bass-Stringer S. Bernardo B. C. May C. N. Thomas C. J. Weeks K. L. McMullen J. R. (2018). Adeno-associated virus gene therapy: translational progress and future prospects in the treatment of heart failure. Heart Lung Circ. 27 (11), 1285–1300. 10.1016/j.hlc.2018.03.005 29703647

[B3] Bish L. T. Morine K. Sleeper M. M. Sanmiguel J. Wu D. Gao G. (2008). Adeno-associated virus (AAV) serotype 9 provides global cardiac gene transfer superior to AAV1, AAV6, AAV7, and AAV8 in the mouse and rat. Hum. Gene Ther. 19 (12), 1359–1368. 10.1089/hum.2008.123 18795839 PMC2940566

[B4] Bolte S. Cordelieres F. P. (2006). A guided tour into subcellular colocalization analysis in light microscopy. J. Microsc. 224 (3), 213–232. 10.1111/j.1365-2818.2006.01706.x 17210054

[B5] Bulcha J. T. Wang Y. Ma H. Tai P. W. L. Gao G. (2021). Viral vector platforms within the gene therapy landscape. Signal Transduct. Target Ther. 6 (1), 53. 10.1038/s41392-021-00487-6 33558455 PMC7868676

[B6] Fischer C. Milting H. Fein E. Reiser E. Lu K. Seidel T. (2019). Long-term functional and structural preservation of precision-cut human myocardium under continuous electromechanical stimulation *in vitro* . Nat. Commun. 10 (1), 117. 10.1038/s41467-018-08003-1 30631059 PMC6328583

[B7] Gabisonia K. Prosdocimo G. Aquaro G. D. Carlucci L. Zentilin L. Secco I. (2019). MicroRNA therapy stimulates uncontrolled cardiac repair after myocardial infarction in pigs. Nature 569 (7756), 418–422. 10.1038/s41586-019-1191-6 31068698 PMC6768803

[B8] Groenewegen A. Rutten F. H. Mosterd A. Hoes A. W. (2020). Epidemiology of heart failure. Eur. J. Heart Fail 22 (8), 1342–1356. 10.1002/ejhf.1858 32483830 PMC7540043

[B9] Inagaki K. Fuess S. Storm T. A. Gibson G. A. McTiernan C. F. Kay M. A. (2006). Robust systemic transduction with AAV9 vectors in mice: efficient global cardiac gene transfer superior to that of AAV8. Mol. Ther. 14 (1), 45–53. 10.1016/j.ymthe.2006.03.014 16713360 PMC1564441

[B10] Kang C. Qiao Y. Li G. Baechle K. Camelliti P. Rentschler S. (2016). Human organotypic cultured cardiac slices: new platform for high throughput preclinical human trials. Sci. Rep. 6, 28798. 10.1038/srep28798 27356882 PMC4928074

[B11] Katz M. G. Fargnoli A. S. Weber T. Hajjar R. J. Bridges C. R. (2017). Use of adeno-associated virus vector for cardiac gene delivery in large-animal surgical models of heart failure. Hum. Gene Ther. Clin. Dev. 28 (3), 157–164. 10.1089/humc.2017.070 28726495 PMC5655838

[B12] Li C. Samulski R. J. (2020). Engineering adeno-associated virus vectors for gene therapy. Nat. Rev. Genet. 21 (4), 255–272. 10.1038/s41576-019-0205-4 32042148

[B13] Liu Z. Klose K. Neuber S. Jiang M. Gossen M. Stamm C. (2020). Comparative analysis of adeno-associated virus serotypes for gene transfer in organotypic heart slices. J. Transl. Med. 18 (1), 437. 10.1186/s12967-020-02605-4 33208161 PMC7673099

[B14] Meki M. H. Miller J. M. Mohamed T. M. A. (2021). Heart slices to model cardiac physiology. Front. Pharmacol. 12, 617922. 10.3389/fphar.2021.617922 33613292 PMC7890402

[B15] Nunez-Toldra R. Kirwin T. Ferraro E. Pitoulis F. G. Nicastro L. Bardi I. (2022). Mechanosensitive molecular mechanisms of myocardial fibrosis in living myocardial slices. Esc. Heart Fail 9 (2), 1400–1412. 10.1002/ehf2.13832 35128823 PMC8934971

[B16] Ou Q. Jacobson Z. Abouleisa R. R. E. Tang X. L. Hindi S. M. Kumar A. (2019). Physiological biomimetic culture system for pig and human heart slices. Circ. Res. 125 (6), 628–642. 10.1161/CIRCRESAHA.119.314996 31310161 PMC6715512

[B17] Palomeque J. Chemaly E. R. Colosi P. Wellman J. A. Zhou S. Del Monte F. (2007). Efficiency of eight different AAV serotypes in transducing rat myocardium *in vivo* . Gene Ther. 14 (13), 989–997. 10.1038/sj.gt.3302895 17251988

[B18] Pitoulis F. G. Watson S. A. Perbellini F. Terracciano C. M. (2020). Myocardial slices come to age: an intermediate complexity *in vitro* cardiac model for translational research. Cardiovasc Res. 116 (7), 1275–1287. 10.1093/cvr/cvz341 31868875 PMC7243278

[B19] Pitoulis F. G. Nunez-Toldra R. Xiao K. Kit-Anan W. Mitzka S. Jabbour R. J. (2021). Remodelling of adult cardiac tissue subjected to physiological and pathological mechanical load *in vitro* . Cardiovasc Res. 10.1093/cvr/cvab084 PMC8859636 33723566

[B20] Prasad K. M. Xu Y. Yang Z. Acton S. T. French B. A. (2011). Robust cardiomyocyte-specific gene expression following systemic injection of AAV: *in vivo* gene delivery follows a Poisson distribution. Gene Ther. 18 (1), 43–52. 10.1038/gt.2010.105 20703310 PMC2988989

[B21] Qiao Y. Dong Q. Li B. Obaid S. Miccile C. Yin R. T. (2019). Multiparametric slice culture platform for the investigation of human cardiac tissue physiology. Prog. Biophys. Mol. Biol. 144, 139–150. 10.1016/j.pbiomolbio.2018.06.001 29960680

[B22] Savarese G. Lund L. H. (2017). Global public Health burden of heart failure. Card. Fail Rev. 3 (1), 7–11. 10.15420/cfr.2016:25:2 28785469 PMC5494150

[B23] Taylor C. J. Ryan R. Nichols L. Gale N. Hobbs F. R. Marshall T. (2017). Survival following a diagnosis of heart failure in primary care. Fam. Pract. 34 (2), 161–168. 10.1093/fampra/cmw145 28137979 PMC6192063

[B24] Tzahor E. Poss K. D. (2017). Cardiac regeneration strategies: staying young at heart. Science 356 (6342), 1035–1039. 10.1126/science.aam5894 28596337 PMC5614484

[B25] Wang D. Tai P. W. L. Gao G. (2019). Adeno-associated virus vector as a platform for gene therapy delivery. Nat. Rev. Drug Discov. 18 (5), 358–378. 10.1038/s41573-019-0012-9 30710128 PMC6927556

[B26] Watson S. A. Scigliano M. Bardi I. Ascione R. Terracciano C. M. Perbellini F. (2017). Preparation of viable adult ventricular myocardial slices from large and small mammals. Nat. Protoc. 12 (12), 2623–2639. 10.1038/nprot.2017.139 29189769

[B27] Zincarelli C. Soltys S. Rengo G. Rabinowitz J. E. (2008). Analysis of AAV serotypes 1-9 mediated gene expression and tropism in mice after systemic injection. Mol. Ther. 16 (6), 1073–1080. 10.1038/mt.2008.76 18414476

[B28] Zincarelli C. Soltys S. Rengo G. Koch W. J. Rabinowitz J. E. (2010). Comparative cardiac gene delivery of adeno-associated virus serotypes 1-9 reveals that AAV6 mediates the most efficient transduction in mouse heart. Clin. Transl. Sci. 3 (3), 81–89. 10.1111/j.1752-8062.2010.00190.x 20590676 PMC3962265

