# Supplementary material for: Living myocardial slices for the study of nucleic acid-based therapies
Source: Front Bioeng Biotechnol. 2023 Oct 24;11:1275945. doi: 10.3389/fbioe.2023.1275945 (PMC10628718; doi:10.3389/fbioe.2023.1275945)
Supplement: Supplementary file 1 [file DataSheet1.PDF]

Supplementary Data

Living myocardial slices for the study of nucleic acid-based therapies

R. Nunez-Toldra, A. Del Canizo, I. Secco, L. Nicastro, M. Giacca, C.M. Terracciano

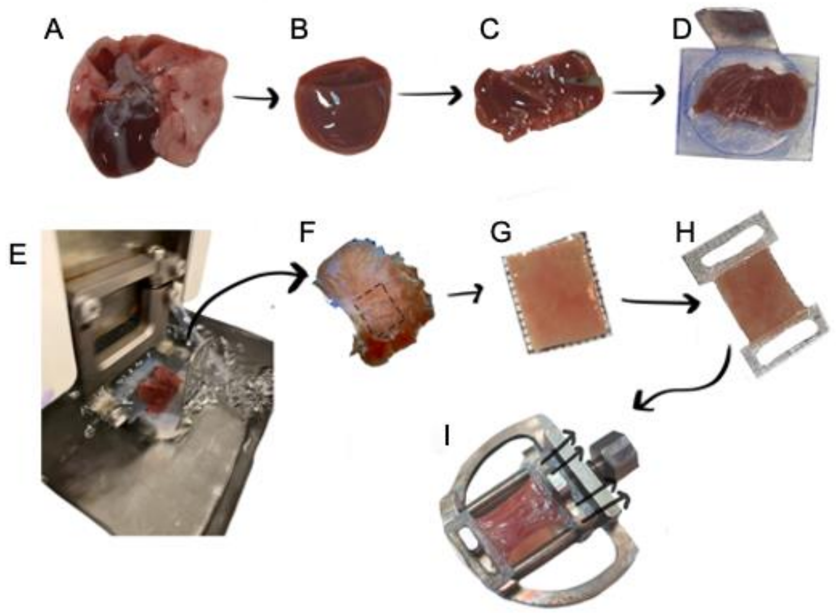

Figure S1. Outline of LMS preparation. (A-B) After heart removal, the surrounding tissues and the atria are removed. (C) Once the heart is completely isolated, an incision is made towards the apex to flatten the tissue. (D-E) The block is then glued to an agarose-embedded holder and immersed in the containing-vibratome bath with Tyrode's solution. (F-G) Once the slice is obtained, an area of approximately 1 cm^2 of fibre alignment is identified and isolated. (H) Rings are glued perpendicularly to fibre alignment allowing the slice to be placed on custom-made stainless-steel stretchers. (I) The stretcher enables to stretch the slice to the desired length in the direction of its fibres.

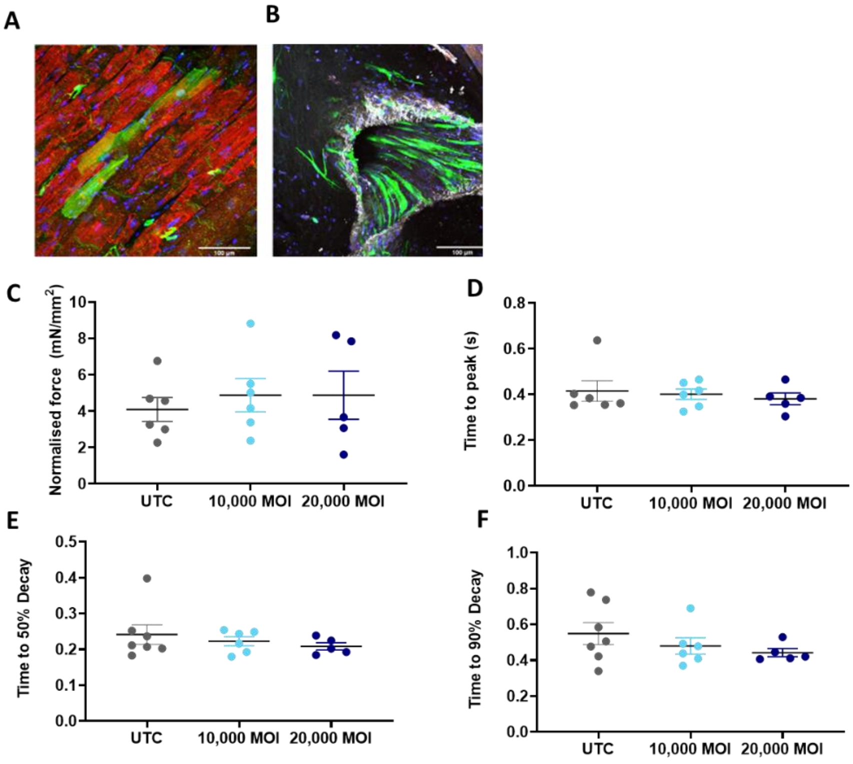

Figure S2. Transduction of Human Failing LMS. Human LMS were transduced with AAV6-pZAC-eGFP and cultured for 72 hours under electrical stimulation. (A) Confocal images of transduced human LMS stained for eGFP (green), α-actinin (red), vimentin (grey) and Hoechst 33342 (blue). (A) Confocal representative images of eGFP positive cardiomyocytes and stromal cells on the surface of LMS. (B) Image of a micro vessel with eGFP positive endothelial cells in the lumen. (C-F) Contractility parameters measured 72h after transduction: (C) Normalilsed Force versus cross sectional area of LMS; (D) Time to reach the peak amplitude of contraction; (E) Time between maximum amplitude of contraction and 50% of the decay; (F) Time between maximum amplitude of contraction and 90% of the decay. Each dot represents an individual LMS (N=2 donors).

Antibody/stain	Origin	Dilution used	Manufacturer	Catalog number
**Primary antibodies**				
Anti–vimentin	Chicken	1:4000	ThermoFisher, US	PA1-10003
Anti-α-actinin	Mouse	1:500	Sigma Aldrich, UK	A7811
Anti-GFP	Rabbit	1:2000	Abcam, UK	ab290
Biotin conjugated anti-isolectin-B4	-	1:400	ThermoFisher, US	I21414
**Secondary antibodies**				
Alexa Fluor 488 anti-rabbit				A21206
Alexa Fluor 488 anti-mouse	Donkey			A21202
Alexa Fluor 555 anti-mouse				A31570
Alexa Fluor 555 anti-rabbit				A21428
Alexa Fluor 647 anti-chicken	Goat	1:2000	ThermoFisher, US	A-21449
Alexa Fluor 647 anti-mouse				A-21235
Alexa Fluor 633 anti-goat	Donkey			A-21082
Alexa Fluor 555 Streptavidin	-	1:1000		S21381

Table S1. Antibodies and concentrations used for immunofluorescence staining.
